# Feasibility Assessment of 3D Printing-Based Tubular Tissue Flap in a Porcine Model for Long Segmental Tracheal Reconstruction

**DOI:** 10.1007/s13770-025-00718-9

**Published:** 2025-05-21

**Authors:** Jeong Hun Park, Nettie E. Brown, Sarah Jo Tucker, Johnna S. Temenoff, Mark El-Deiry, Hyun-Ji Park, Andrew T. Tkaczuk, Scott J. Hollister

**Affiliations:** 1 Wallace H. Coulter Department of Biomedical Engineering, Georgia Institute of Technology and Emory University, 313 Ferst Drive, Atlanta, GA 30332 USA; 2 Center for 3D Medical Fabrication, Georgia Institute of Technology, 313 Ferst Drive, Atlanta, GA 30332 USA; 3 Global Center for Medical Innovation, 575 14th Street, Atlanta, GA 30318 USA; 4 Petit Institute for Bioengineering and Bioscience, Georgia Institute of Technology, 315 Ferst Drive, Atlanta, GA 30332 USA; 5 Department of Otolaryngology Head and Neck Surgery, Emory University School of Medicine, 550 Peachtree Street NE, Atlanta, GA 30308 USA; 6 Department of Molecular Science and Technology, Ajou University, 206 Worldcup-Ro, Suwon, 16499 Republic of Korea; 7 Advanced College of Bio-Convergence Engineering, Ajou University, 206 Worldcup-Ro, Suwon, 16499 Republic of Korea

**Keywords:** 3D printing, Selective laser sintering, Tracheal reconstruction, Vascularization, Flap reconstruction

## Abstract

*****BACKGROUND***:**

Despite advances in tissue engineering, current clinical reconstructive options for long segment tracheal defects are limited. In this study, a 3D printing based tubular tissue flap strategy was developed for long segment tracheal reconstruction.

*****METHOD***:**

A stent-patterned airway scaffold with sufficient radial rigidity and longitudinal bending flexibility was designed and its mechanical behavior was analyzed using finite element analysis (FEA). The stent-patterned airway scaffolds with a removable central core to preserve an internal lumen were created by selective laser sintering (SLS) based 3D printing. The stent-patterned airway scaffold with the central core, filled with poly (ethylene glycol) diacrylate-dithiothreitol (PEGDA-DTT) hydrogel containing erythropoietin (EPO) to enhance vascularization, was then implanted into the latissimus dorsi muscle of a Yucatan minipig.

*****RESULTS***:**

A tubular tissue flap, with controlled luminal layer thickness was successfully created by removing the central core from the retrieved tissue flap containing the airway scaffold after 45 days of implantation in the Yucatan minipig model.

*****CONCLUSION***:**

The current work validated the potential of the tubular tissue flap based on the 3D printing as a clinically viable tissue engineering strategy for long segment tracheal reconstruction.

## Introduction

Long segmental tracheal defects present a significant reconstructive challenge. In adults, lesions encompassing less than 50% of the tracheal length and in children, defects less than 40% can be reconstructed with end-to-end anastomosis. Previous interventions that reduce tracheal blood supply including multiple operations, scarring and radiation may complicate reconstruction and reduce the lengths that can be reconstructed using end to end anastomosis. Attempting end-to-end anastomosis for longer segments or in the scenario of revision surgery increases the tension at the anastomosis site, which can lead to local necrosis, dehiscence, and potentially stricture and restenosis [1].

Despite advances in tissue engineering, current clinical reconstructive options for long segment tracheal defects are limited. Allograft implantation of processed cadaveric trachea in pediatric patients requires stenting and has had limited success [2]. Poor viability of the transplanted tissues as well as cartilaginous degradation have been frequently reported in cadaveric tracheal allografts [2–4]. Clinical trials of a decellularized human donor trachea and the recipient’s autologous cells have been made; however, the post-complications were severe with restenosis and collapse of the trachea [5]. Survivability of diseased donor transplanted tracheal allografts have been reported in animal models and recently applied in humans [6, 7]. However, not all individuals are candidates for this option [8–10]. Meanwhile, vascularized autogenous tissue has shown significant reconstructive potential over several studies [11–14]. These vascularized reconstructions require a mechanical support to prevent collapse of the tissue flap and both synthetic and autologous materials were employed [15, 16]. The synthetic materials have been limited to short distance reconstruction and the autologous materials require a separate harvest site with associated risks and potential morbidity to the patient.

**Fig. 1:**
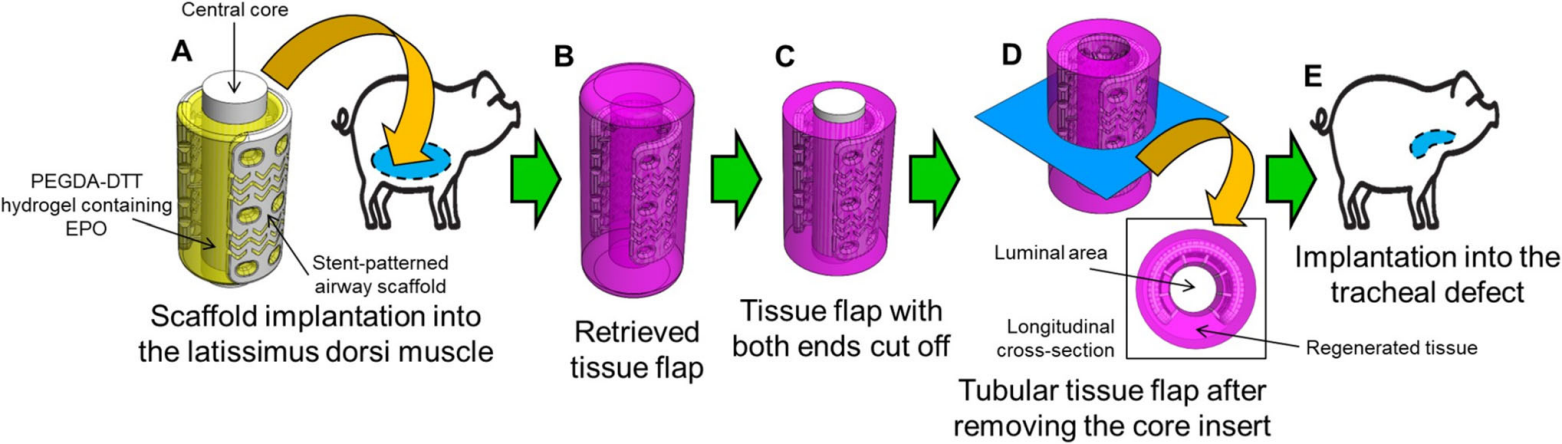
Schematics of 3D printing based tubular tissue flap strategy for long segment tracheal reconstruction. **A** Implantation of the airway scaffold including the central core (white color) and PEGDA-DTT hydrogel containing EPO (yellow color) into the latissimus dorsi muscle of a Yucatan minipig, **B** Retrieval of cylindrical tissue flap composed of the airway scaffold and regenerated tissues, **C** Incision of both ends of the regenerated tissues surrounding the airway scaffold, **D** Removal of the central core from the tissue flap. **E** Implantation of the tubular tissue flap into the segment tracheal defect

Since the first personalized external airway support device (ASD) was developed based on 3D printing, it has been successfully applied to the patients for treatment of life threatening tracheobronchomalacia (TBM) over the last decade [17, 18]. In this study, we aimed to develop a rational tissue flap strategy based on an advanced ASD for long segment tracheal reconstruction. As the framework of a pre-vascularized tubular tissue flap, an airway scaffold incorporating stent-patterns was designed to achieve both sufficient radial rigidity and longitudinal bending flexibility based on the previous ASD. As a release vehicle for erythropoietin (EPO), an angiogenesis stimulating growth factor, poly (ethylene glycol) diacrylate (PEGDA) was combined with dithiothreitol (DTT) to enhance vascularization by promoting hydrolytic degradation. For feasibility test of the pre-vascularized tubular tissue flap, the airway scaffold with the core insert was infilled with PEGDA-DTT hydrogels encapsulated with 0.24 mg/mL of EPO and implanted into the latissimus dorsi muscle in a minipig model (Fig. 1). After 45 days of implantation, we assessed the reconstructed pre-vascularized tubular tissue flap at a proof-of-concept level.

## Materials and methods

### Materials

Poly-ε-caprolactone (PCL, Polyscience Inc., USA) was cryogenically milled, sieved, and mixed with 4% (w/w) hydroxyapatite (HA, Plasma Biotal Ltd., UK) for selective laser sintering (SLS) based 3D printing of the stent-patterned airway scaffold with and without the central core. Polyethylene glycol (PEG), acryloyl chloride (AcCl), Triethylamine (TEA), dithiothreitol (DTT), ammonium persulfate (APS), N,N,N′,N′-tetramethylethylenediamine (TEMED), and potassium chloride (KCl) were purchased from Sigma Aldrich Inc. (St. Louis, MO, USA). EPO was purchased from Creative Biomart (Shirley, NY, USA).

### Airway scaffolds design and finite element simulation

**Fig. 2:**
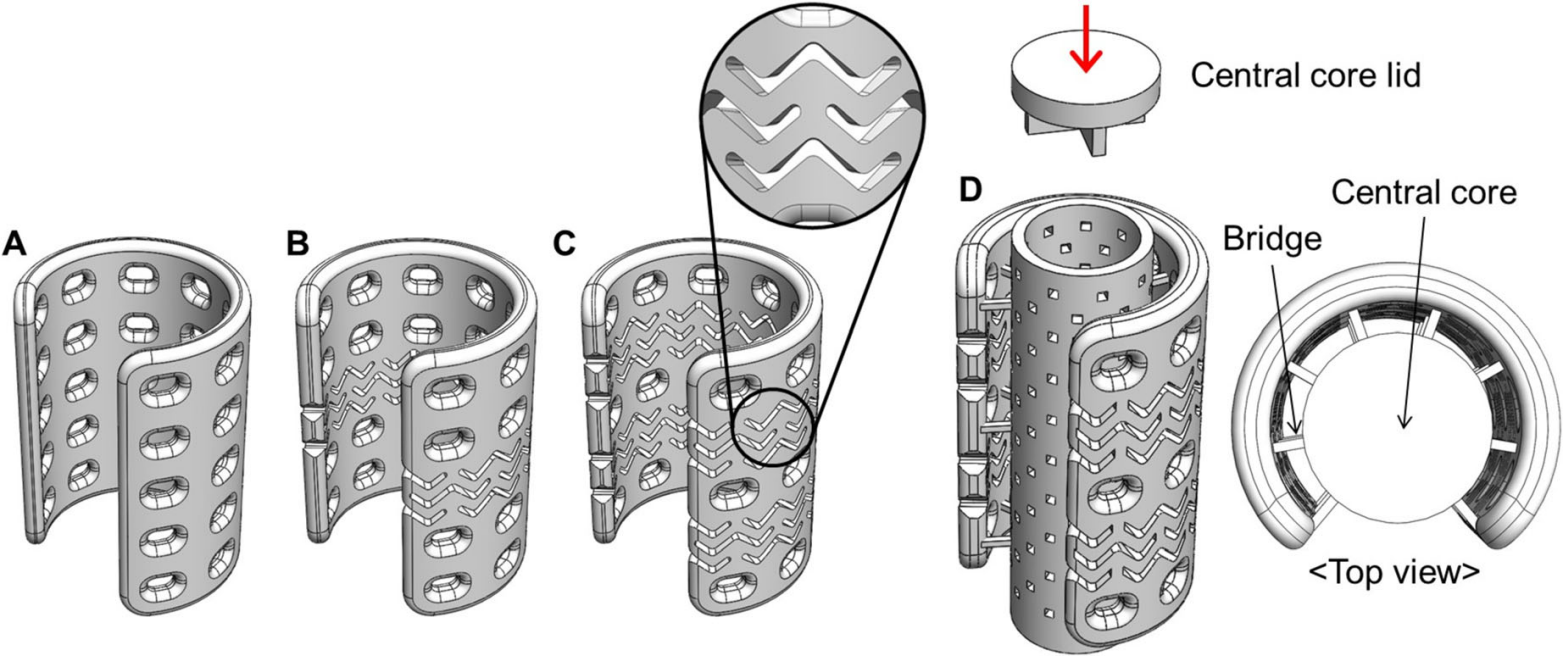
Design of the airway scaffolds. **A** Airway scaffold without stent-pattern, **B** Airway scaffold with a stent-pattern, **C** Airway scaffold with two stent-patterns, **D** The stent-patterned airway scaffold with the hollow porous central core to create the tubular tissue flap *in vivo*

The airway scaffold as a framework for the pre-vascularized tubular tissue flap was designed based on the previous implantable ASD [19]. A stent-pattern, consisting of a series of interconnected sinusoidal networks, was incorporated into the wall of the airway scaffold to enhance its bending flexibility [20–23]. Two different stent-patterned airway scaffolds were designed with 2.3 mm wall thickness while normal airway scaffold, without the stent-pattern, has a 2.0 mm wall thickness (Fig. 2A–C). All airway scaffolds had 32 mm length, 15 mm inner diameter, and 90° opening angle. The central core was added to the luminal area of the airway scaffold to restrict excessive tissue infiltration into the luminal area for tubular tissue flap creation *in vivo*. The central core of a 11 mm diameter was connected to the scaffold wall by a number of bridges having a square cross-section of 500 × 500 µm^2^ (Fig. 1D). This central core is a hollow porous cylindrical structure with a 1 mm wall thickness and 500 µm pores to incorporate PEGDA hydrogel containing EPO. The central core lid is assembled into the luminal area of the central core to prevent leakage of the PEGDA hydrogel after implantation.

FE simulations on radical compression and three-point bending were performed with two different directions (parallel and perpendicular to the scaffold opening) using FEBio studio version 1.6.0 (Febio.org) [24]. 4-node tetrahedral elements were used in the airway scaffold models (without a central core), and the base PCL material for airway scaffolds was considered linear isotropic elastic with a Young’s modulus of 0.116 GPa and a Poisson’s ratio of 0.3 in simulation.

### SLS based 3D printing of the airway scaffolds

The stent-patterned airway scaffolds with and without the central core were created using a Formiga P110 laser sintering system (Electro-Optical Systems (EOS) GmbH, Krailing, Germany). STL files exported from SolidWorks® were imported into Magics software and processed by duplications, translations, rotations, and nesting into labeled sinter boxes on the platform. PSW software (Version 3.6, EOS GmbH) was used to slice the processed STL files into the 100 µm thickness layers. The sliced data was then transferred to the Formiga P110 and the airway scaffolds were created through a laser sintering process using 4 W laser with a scanning speed of 1,500–2,000 mm/sec.

### Mechanical test

A 5944 Single Column mechanical testing system (Instron Corp., Norwood, MA, USA) with a 2kN load cell was used for compression tests of stent-patterned airway scaffolds. Compression at 5 mm/min was applied along the parallel and perpendicular directions to the airway scaffold opening and load–displacement responses were recorded during the tests.

### Hydrogel preparation

Polyethylene glycol diacrylate (PEGDA) was synthesized as previously described [25]. PEG (3.4 kD) was dissolved in dichloromethane (DCM, VWR, Pennsylvania, USA) and reacted with AcCl in an 8:1 AcCl to PEG molar ratio. TEA was added dropwise to catalyze the reaction in a 1:1 TEA to AcCl molar ratio to yield linear PEGDA. PEGDA was separated into an organic phase using potassium chloride (VWR, USA) and precipitated in diethyl ether (Sigma, USA). Solid PEGDA was recovered under vacuum filtration, air dried, and purged with nitrogen before storing at − 20 °C. PEGDA was analyzed via proton NMR (1H NMR) to determine extent of diacrylation. To form hydrogels, 15 wt.%/v PEGDA and 20 mol.% DTT, relative to PEGDA, were mixed with phosphate-buffered saline (PBS, ThermoFisher, MA, USA). The solution was incubated at 37 °C for 30 min to allow for Michael-Type addition of the DTT with PEGDA. 2 mL of a 0.5 mg/mL solution of EPO was added to the hydrogel solution. PBS was added to achieve a total volume of 4.2 mL. 0.022M ammonium persulfate (APS) and 0.022M TEMED were added to initiate crosslinking. All hydrogel components were sterilized using sterile syringe filters (0.45 μm, Sigma, USA) before mixing.

### EPO release test

5624.10 IU (International Units) of EPO was added to the hydrogel solutions before crosslinking. 50 μL of polymerizing hydrogel solutions were placed in cylindrical Teflon molds (6 mm diameter and 1 mm height) for 18 min at 20 °C. After crosslinking, samples were placed in 500 μL PBS at 37 °C on a shaker plate at 65 rpm (Barnstead Lab-Line Shaker, Dubuque, IA) for 3 h. The percent loaded of EPO was then calculated using Eq. 1. In this equation. the amount of EPO in the supernatant at 3 h is defined as “non-loaded”.

1 $$ \% \;Loaded = \left( {\frac{{Initially\;loaded\;\left( {5624.10 IU} \right) - Non - loaded\;\left( {IU} \right)}}{{Initially\;loaded\;\left( {5624.10 IU} \right)}}} \right) \times 100 $$

Supernatant from each sample was removed at each timepoint (Day 1,4, 7, 11, 14, 17, 21, and 28) and replaced with fresh PBS at each timepoint. Release was determined by measuring amount of EPO in the supernatant using an enzyme-linked immunosorbent assay (ELISA, EPO DuoSet, RnD Systems, Minneapolis, MN, USA), as per manufacturer’s instructions, and read with a UV–vis spectrometer (SpectraMax M2, Molecular Device, San Jose, CA) at 450 nm (n = 3). Using a standard curve, cumulative release percent was calculated as in Eq. 2 for each timepoint.

2 $$ Cumulative\;Release\; \left( \% \right) = \left( {\frac{{Sum\;of\;release\;\left( {Day 0 \to timepoint\;\left( {IU} \right)} \right)}}{{Quantity\;loaded \left( {IU} \right)}}} \right) \times 100 $$

### Implant preparation and implantation

**Fig. 3:**
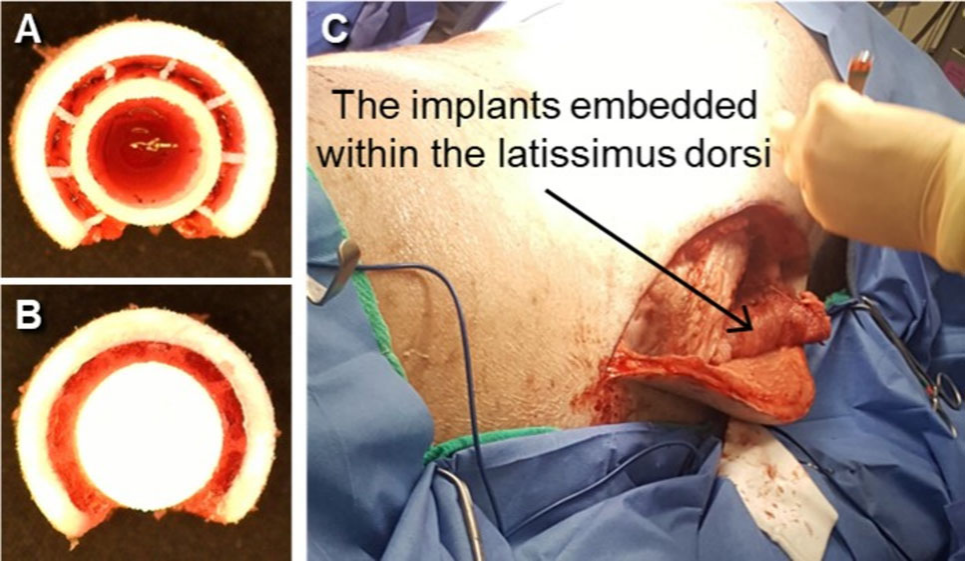
The top view of the airway scaffold infilled with PEGDA hydrogel containing EPO **A** before and **B** after the central core lid assembly, **C** The latissimus dorsi musculature embedding the airway scaffold infilled with PEGDA hydrogel containing EPO

The sterilized two stent-patterned airway scaffold with the central core was placed into the custom mold. Immediately after mixing all hydrogel components, precursor solutions were injected into the mold to fill the central core as well as the gap between the airway scaffold and central core. The central core was then assembled after PEGDA crosslinking for 20 min at room temperature (Fig. 3A, B).

Institutional Animal Care and Use Committee (IACUC) approval was obtained from the Translational Training and Testing Labs, Inc. (T3 Labs), and humane care of each animal was performed during the implantation under the care of a certified veterinarian to ensure standard intraoperative anesthesia care and monitoring. The animal was provided a pre-incisional antibiotic (Baytril at 5–6 mg/kg), anesthetized with a combination of (Ketamine 15–16 mg/kg and Xylazine 1–2 mg/kg and orotracheally intubated. Anesthetic maintenance included a combination of Isoflurane (0–5%) inhaled anesthetic and analgesics.

The implants were embedded within the latissimus dorsi musculature of a single Yucatan pig, one per side (Fig. 3C). The right and left flank of the animal was sterilized with Iodophor and draped. A curvilinear incision was made medially along a sagittal plane over the underlying latissimus musculature. Cutaneous flaps were elevated to expose the belly of the latissimus dorsi. Spinal and costal attachments were dissected freely. Subsequently, the scaffold was individually circumferentially embedded and sutured within the belly of the musculature leaving the lumen of the scaffold patent. The same approach was performed on the contralateral side with the same scaffold conditions. The wounds were copiously irrigated and subsequently closed in layers using absorbable Vicryl sutures and deep and staples for the skin. The pig was recovered from anesthesia and the animal was monitored for 45 days to allow time for tissue integration as well as evidence of postoperative complications. Postoperative analgesia was maintained with Rimadyl (3–4 mg/kg) and Buprenorphine (0.005–0/01 mg/kg). Postoperative antibiotics were continued with Baytril (5–6 mg/kg) SID for 10 days. At 45 days after implantation, the animal was euthanized with Euthasol (1 mL/5 kg) and necropsy was performed to harvest the latissimus musculature with scaffolds in situ to allow histological assessment.

### Histological analysis

A Yucatan minipig was sacrificed and the implants were retrieved for histological analysis at 45 days after implantation. Samples were fixed with 10% neutral buffered formalin (NBF) and processed with a TP1020 tissue processor (Leica Biosystems, Wetzlar, Germany) to prepare tissue-embedded paraffin blocks. Blocks were cut into 8 μm thick sections and the tissue sections were stained with hematoxylin & eosin (H&E) and Masson’s trichrome (MT). Stained tissue sections were scanned using an Olympus Nanozoomer whole slide scanner (Hamamatsu Photonics, Hamamatsu, Japan) and the scanned images were analyzed using QuPath software (version 0.3.2, https://qupath.github.io/).

### Statistical analysis

All experiments were performed at least three times and quantitative data were represented as the mean ± SD. Statistical analysis was performed using two-tailed Student’s t test to determine significant difference between two experimental groups. A *p*-value less than 0.05 was considered to be statistically significant.

## Results

### Design and mechanical behavior of airway scaffolds

**Fig. 4:**
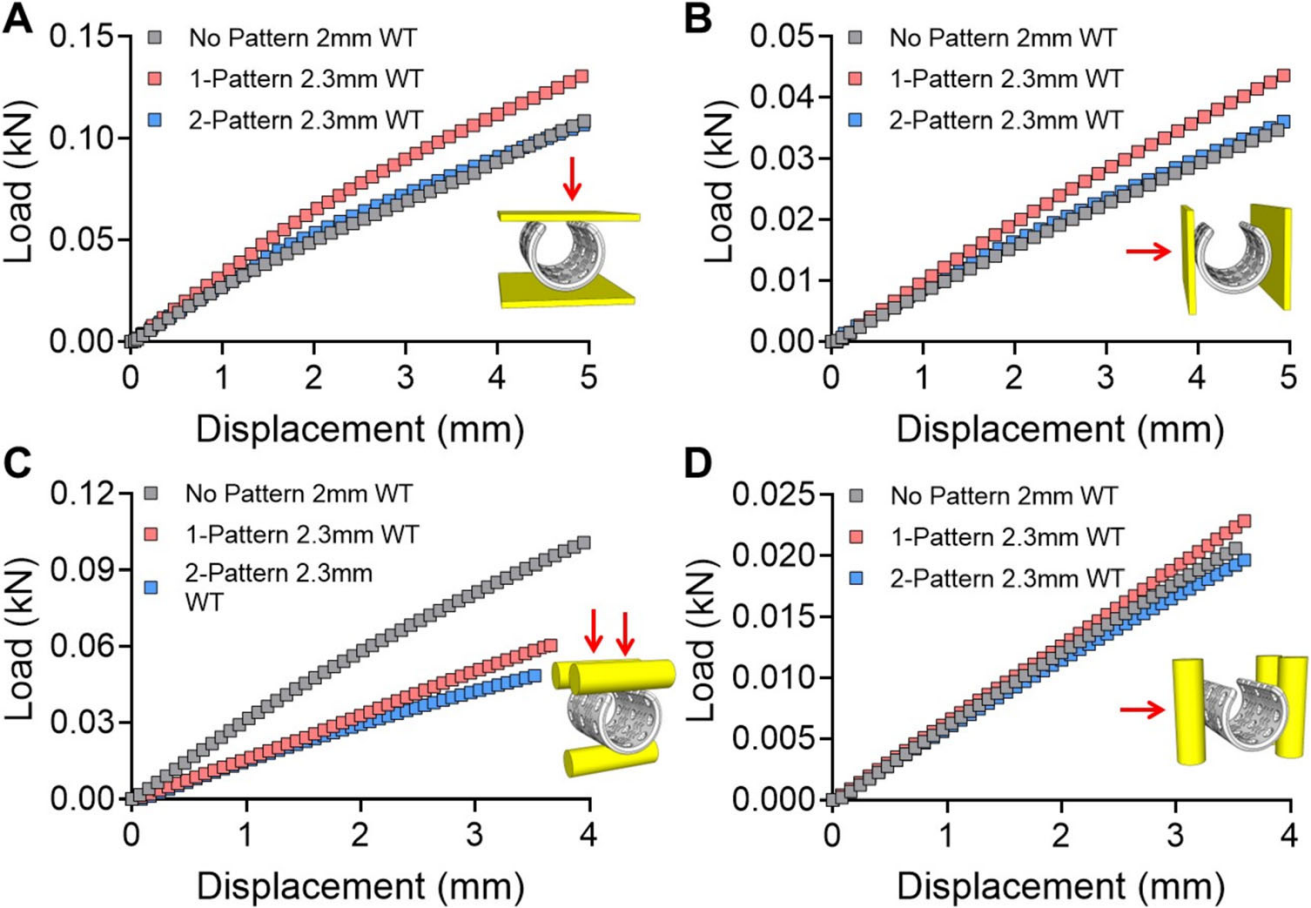
Mechanical behavior analysis of airway scaffolds according to the stent-pattern application. The load and displacement curves of airway scaffolds under **A** parallel compression, **B** perpendicular compression, **C** parallel three-point bending, and **D** perpendicular three-point bending

Three different airway scaffolds were designed and the effect of stent patterns on the mechanical behavior of airway scaffolds was analyzed through FE simulation. As a control, the normal airway scaffold without a pattern was designed with a wall thickness of 2 mm, the same as that of the previous implantable ASD. This wall thickness was determined for the ASD printed using SLS to provide sufficient radial stiffness to withstand forces exerted by the external surrounding tissues *in vivo* [26–30]. Two stent-patterned airway scaffolds were then designed by incorporating different numbers of stent patterns into the ASD. In addition, the wall thickness of two stent-patterned airway scaffolds increased to 2.3 mm to compensate for the decrease in radial stiffness caused by the incorporation of stent patterns. FEA results showed that the airway scaffold with a single stent-pattern exhibited the highest radial stiffness, while the airway scaffold with two stent-patterns had almost the same radial stiffness as the normal airway scaffold. (Fig. 4A, B). The airway scaffold with two stent-patterns demonstrated the highest bending flexibility in both parallel and perpendicular directions despite its thicker wall thickness, as indicated by its lower bending stiffness (Fig. 4C, D). Although the airway scaffold with a single stent-pattern exhibited higher bending flexibility than the normal airway scaffold in the parallel direction, it was stiffer than the normal airway scaffold in the perpendicular direction due to its increased wall thickness. Based on these results, the airway scaffold with two stent-patterns was selected for further *in vivo* study as it offered radial stiffness comparable to that of the normal airway scaffold while exhibiting the highest bending flexibility.

### 3D printed airway scaffold with 2 stent-patterns

**Fig. 5:**
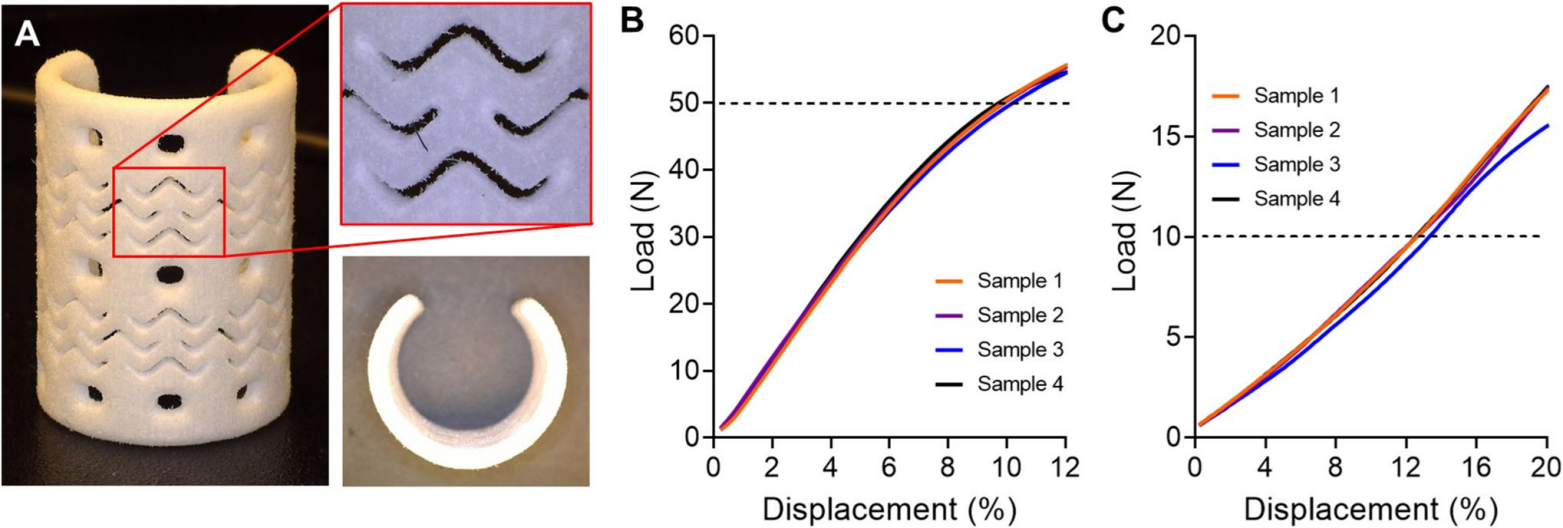
3D Printing and mechanical test results of the airway scaffold with two stent-patterns. **A** Photographs of the printed airway scaffold with two stent-patterns. The load and displacement curves of airway scaffolds under **B** parallel compression and **C** perpendicular compression to the airway scaffold opening (n = 4)

The airway scaffold with two stent-patterns was successfully fabricated by SLS based 3D printing for *in vivo* study (Fig. 5A). The suture holes as well as stent-patterns in the wall were clearly visible. The compression test results demonstrated that the printed airway scaffold with two stent-patterns met the mechanical design requirements of the previous ASD, allowing less than 40–50% of gap between the platens under 40–50 N in parallel compression and 20% of opening angle gap under 10 N in perpendicular compression, respectively (Fig. 5B, C) [31, 32].

### Release of EPO

**Fig. 6:**
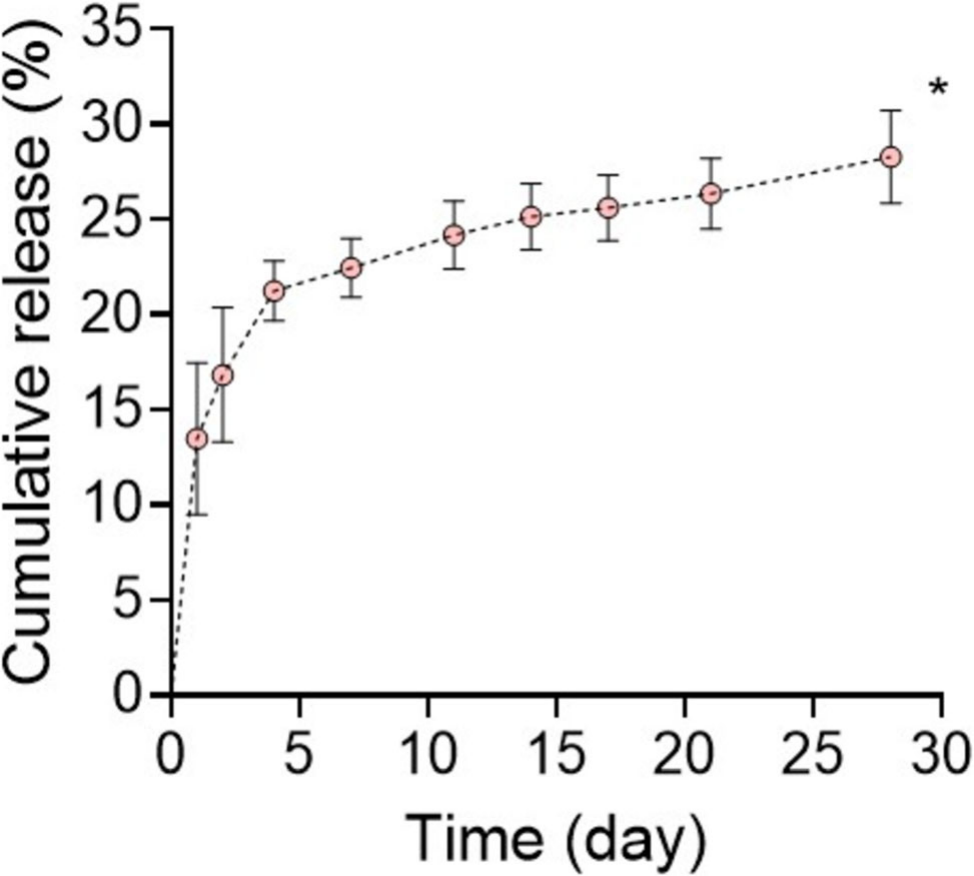
Cumulative release of EPO from PEGDA hydrogel (n = 3, *p < 0.05 versus day 1)

EPO exhibited the following release profile from the hydrogels: 13.47% (Day 1), 16.81% (Day 2), 21.22% (Day 3), 22.44% (Day 7), 24.17% (Day 11), 25.13% (Day 14), 25.60% (Day 17), 26.33% (Day 21), and 28.26% (Day 28), demonstrating sustained release over 28 days *in vitro* (Fig. 6). A cumulative release of EPO from PEGDA-DTT hydrogel over 28 days *in vitro* is also corelated with previous studies [33, 34].

### Vascularized tubular tissue flap

**Fig. 7:**
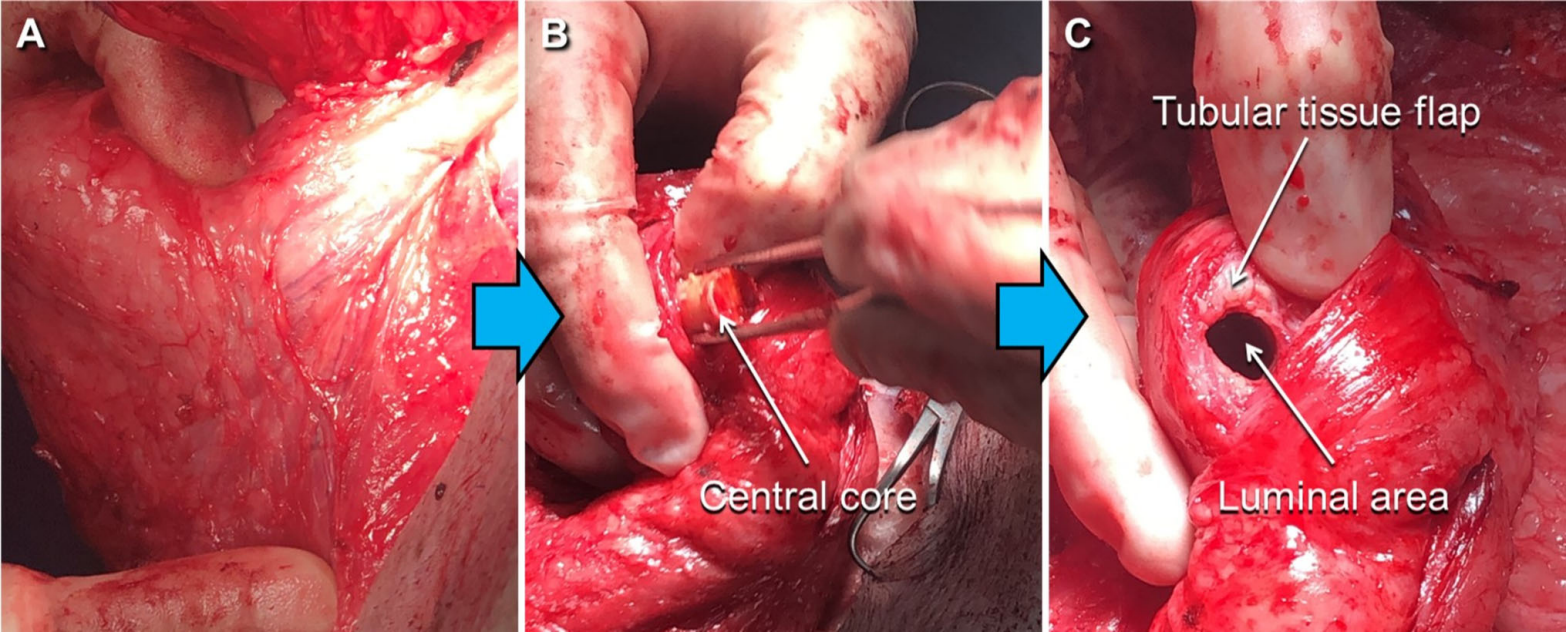
Pedicled latissimus dorsi tubular tissue flap after 45 days of implantation. **A** Incision of the muscle tissue flap with the embedded airway scaffold, **B** Central core removal from the tissue flap, **C** The tubular tissue flap based on the airway scaffold after removal of the central core

The pedicled latissimus dorsi flap had robust and expected viability after the harvest, implant placement, and flap delay for reconstructive applications at 45 days after implantation due to a favorable axial blood supply (Fig. 7A). The central core was successfully removed from the reconstructed tissue flap during retrieval, and the vascularized tubular tissue flap embedding the airway scaffold was readily created (Fig. 7B, C). After removal of the central core, no necrotic material or granulation tissue within the lumen of the tissue flap was observed. This confirmed that the stent-patterned airway scaffold was well integrated with no evidence of infection.

**Fig. 8:**
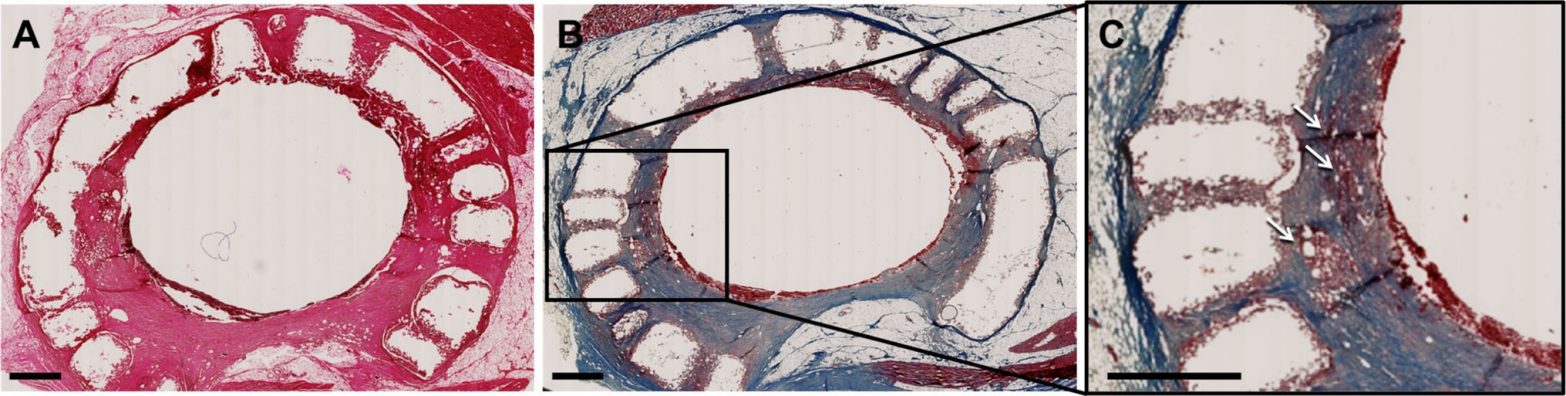
Evaluation of tissue formation surrounding the airway scaffold. **A** H&E and **B** MT staining results of longitudinal cross-section of the regenerated tubular tissue flap based on the airway scaffold at 45 days after implantation. Scale bar, 2 mm. White arrows indicate the blood vessels in the regenerated luminal tissue

H&E image of longitudinal cross-section of the tubular tissue flap indicated that the luminal surface of the airway scaffold was completely covered by reconstructed tissue and the airway scaffold was incorporated with reconstructed surrounding tissues within 45 days (Fig. 8A). Muscle tissue formation with muscle fibers (red) and collagen (blue) was also confirmed by MT staining result (Fig. 8B). Infiltrated microvessels were also found in the reconstructed muscle tissue around the airway scaffold (Fig. 8C).

## Discussion

Long segment tracheal replacement remains a clinical challenge as traditional segmental resection and anastomosis are limited even with cervical and mediastinal release maneuvers. More recent methods of tracheal replacement have focused on transplantation and tissue engineering [35, 36]. Given the complexities of a segmental vascular supply, the ideal solution would be an engineered replacement with biomimetic, biomechanical, and biocompatible properties. This would also minimize the need for long-term immunosuppression, which increases the risk of tumor recurrence in malignant disease, a not uncommon reason for tracheal resection. In this context, an engineered tissue flap strategy based on 3D printing could be expected to prove to be a means to circumvent some of these issues.

The engineered tissue flap for long segment tracheal reconstruction should have sufficient bending flexibility as well as radial stiffness to maintain the patency and stability, similar to the native trachea. The airway scaffold used as a framework for the tubular tissue flap in this study features an advanced design from the previous ASD. The ASD is an open tubular structure, with a plurality of suture holes in its wall, specifically designed to provide sufficient radial stiffness for external support of airway defects but without considering the bending flexibility of the native trachea. The airway scaffold used in this study retained the same form of the open tubular structure as the ASD; however, it incorporated the geometric patterns of the stent into its wall, which is commonly used for treating narrowed or blocked blood vessels. The stent pattern enhanced longitudinal bending flexibility of the airway scaffold by allowing it to deform more easily while ensuring structural patency when it is bent [21, 23]. This enhanced bending flexibility enabled the airway scaffold to be more adaptable to the dynamic mechanical environment of a long segment of the trachea by ensuring mechanical stability and patency for long segmental tracheal lesion.

The stent-pattern is particularly of significant importance in enhancing bending flexibility. The number of the stent-patterns applied to the airway scaffold depends on its longitudinal length, which corresponds to the anticipated length of the tracheal defect. While increasing number of stent-patterns enhanced bending flexibility of the airway scaffold, it also adversely affects radial stiffness, which is crucial for maintaining patency after implantation for tissue flap creation and subsequent tracheal reconstruction. Therefore, the wall thickness of the stent-patterned airway scaffold becomes an additional key design parameter to achieve enhanced bending flexibility without compromising the radial patency. In this study, the stent-patterned airway scaffolds of a 2 mm wall thickness exhibited insufficient radial stiffness compared to the normal airway scaffold of the same wall thickness (data now shown); however, it was readily addressed by increasing the wall thickness to 2.3 mm. Mechanical test results also confirmed that the printed stent-patterned airway scaffold successfully met the design requirements of the ASD, which were clinically established based on arterial pressure from aorta and innominate arteries, as well as surrounding thoracic tissue [26–30]. Consequently, when applied in the same manner as the previous ASD, the stent-patterned airway scaffold is expected to be able to address longer stenotic segments than previous ASD by its enhanced longitudinal bending flexibility and sufficient radial stiffness, which ensure mechanical stability and patency for long segmental lesion following external implantation around extensive segmental defects.

In this study, we assessed the potential of the airway scaffold as a framework for the pre-vascularized tubular tissue flap for reconstruction of extended tracheal defects as in the other application. In addition to the advanced design of airway scaffold, a PEGDA-DTT hydrogel containing EPO was infilled into the airway scaffold with central core to provide a soft-tissue interface and facilitate EPO delivery. EPO, an FDA-approved drug, promotes angiogenesis and enhances the formation of functional and mature blood vessels [37, 38]. Beyond its angiogenic properties, EPO enhances pericyte recruitment, which is crucial for stabilizing newly formed blood vessels and ensuring their long-term functionality. Compared to vascular endothelial growth factor (VEGF) and other growth factors, EPO exhibits a stronger anti-inflammatory profile, reducing pro-inflammatory cytokine production and fostering a regenerative environment [38]. In addition, EPO has been reported to be more effective in initiating robust vascularization while other growth factors including angiopoietin-1 (ANG-1) and platelet-derived growth factor (PDGF) play a more significant role in later stages of vascular development [39, 40].

5624.10 IU (International Units) of EPO was used in this study as doses within the range of 5000–6000 IU provide significant pro-angiogenic effects without triggering adverse systemic effects such as excessive erythropoiesis or thrombosis [37]. This dosage ensures sufficient activation of endothelial cells and recruitment of pericytes, promoting stable and functional blood vessel formation around the hydrogel and tracheal splint. Encapsulation of EPO in a PEGDA hydrogel allows for localized, sustained release, reducing systemic exposure and minimizing the risk of side effects like polycythemia [38, 41]. Lower doses (< 3000 IU) often fail to achieve sufficient vascularization, while excessively high doses (> 10,000 IU) increase the risk of systemic complications. The dosage is customized for the latissimus dorsi muscle, a well-vascularized tissue that supports implant integration. This ensures that the EPO concentration is appropriate to stimulate endothelial cells and support neovascularization within the local microenvironment [38].

The airway scaffold with stent-patterns was created using SLS-based 3D printing of PCL, which has been used to produce ASD for clinical translations under the U.S. Food and Drug Administration (FDA) Expended Access [18, 42, 43]. Despite the clinically proven biocompatibility of PCL, the first preliminary test of the stent-patterned airway scaffold without the central core yielded unsatisfactory results, specifically aggregation of blood products and cellular debris in the luminal area without tubular tissue formation following implantation into the caudal portion of each latissimus dorsi in a pig model (data not shown). An anatomic assessment of the pig cadaver subsequently confirmed that geometrically a latissimus rotational flap would be superior to free flap reconstruction due to pedicle access as well as a limited availability of reliable external carotid artery branch access within the neck. In addition, the removable central core was then implemented to limit the aggregation of blood products and cellular debris in the luminal area of the airway scaffold.

In the second trial, the stent-patterned airway scaffold with central core successfully created a vascularized autogenous tubular tissue flap within 45 days. The central core was readily removed from the luminal area of the airway scaffold and the formation of the tubular tissue flap with uniform luminal thickness was achieved. The hydrogel formulation of 20 mol.% ratio of DTT to PEGDA was determined to achieve hydrolytic degradation within 1 month since 10–30% molar ratios of DTT to PEGDA achieved hydrolytic degradation between 2 and 4 weeks *in vitro* in the previous studies [25, 34, 44]. The histology results showed a lack of hydrogel, confirming that the PEGDA-DTT hydrogel had degraded within 45 days *in vivo*, consistent with previous *in vitro* results [45]. As PEGDA degrades, it provides space for stromal tissue infiltration, which is critical for implant integration and long-term function. Stromal cells contribute to extracellular matrix (ECM) deposition and provide structural support for newly formed vessels, ensuring their stability [46]. PEGDA degradation products can influence the recruitment and activation of inflammatory cells (e.g., macrophages and neutrophils). A mild, transient inflammatory response is beneficial for angiogenesis, as macrophages release VEGF, FGF, and other pro-angiogenic factors [47, 48]. However, excessive or prolonged inflammation due to degradation products or improper hydrogel design could lead to fibrosis or implant rejection, compromising vascularization and tissue integration. Encapsulated EPO mitigates excessive inflammation by reducing the release of pro-inflammatory cytokines and polarizing macrophages toward a reparative M2 phenotype [37]. This helps create a microenvironment favorable for vascularization and tissue recruitment despite hydrogel degradation. In addition, a gradual release ensures that EPO levels remain within the therapeutic window throughout the critical early stages of vascularization [37]. A release rate of 25% over 4 weeks prevents the sharp concentration spikes associated with bolus administration, which could lead to receptor oversaturation and suboptimal vessel formation. EPO’s half-life *in vivo* is relatively short when administered systemically. Encapsulation in the PEGDA hydrogel provides a mechanism for sustained delivery, ensuring local availability for vascularization over an extended period [38]. The 4-week period corresponds to the critical window during which early vessel formation transitions into vessel stabilization and maturation, aligning with the biological timeline of angiogenesis [41]. In addition, In the latissimus dorsi muscle of a pig, the vascular bed is extensive, but the local tissue volume requires gradual factor release to maintain consistent exposure across the implant site. Therefore, this slow diffusion over 4 weeks ensures uniform diffusion and availability [38].

Although the vascularized tubular tissue flap was successfully created using 3D printing-based stent-patterned airway scaffold, we identified further issues to be addressed. There are potential pitfalls with such a tracheal replacement device. Of physiological concern is limitations in secretory behavior and mucociliary clearance due to absent respiratory epithelium in the luminal surface of the tubular tissue flap. This was not assessed in situ; however, it has been reported that in individuals with an adequate cough this consequence can be manageable [49]. Furthermore, the latissimus dorsi rotational flap embedding the airway scaffold was to be anastomosed into a segmental tracheal defect with a planned tracheotomy for airway protection. During this portion of the procedure, it was determined that an extensive and non-survivable surgical approach would be required for geometric access to the vascular pedicle to ensure adequate degrees of freedom for flap rotation and inset. It was therefore decided to abort this procedure to continue scaffold maturation to investigate long-term tissue integration for 45 days.

In conclusion, we developed the tubular tissue flap strategy based on 3D printing for long segment tracheal reconstruction. The stent-pattern was introduced to increase bending flexibility of the airway scaffold, which is critical for the application to long segment tracheal defects. The vascularized tubular tissue flap with controlled luminal wall thickness was successfully created based on the stent-patterned airway scaffold with a central core. Future work will focus on the long segment tracheal reconstruction using the tubular tissue flap in terms of tracheal epithelial regeneration on the luminal surface.

## Data Availability

The datasets used and/or analyzed during the current study are available from the corresponding author on reasonable request.
